# Innate Immunity and Inflammation Post-Stroke: An α7-Nicotinic Agonist Perspective

**DOI:** 10.3390/ijms161226141

**Published:** 2015-12-04

**Authors:** Silke Neumann, Nicholas J. Shields, Thomas Balle, Mary Chebib, Andrew N. Clarkson

**Affiliations:** 1 Department of Pathology, University of Otago, Dunedin 9054, New Zealand; silke.neumann@otago.ac.nz (S.N.); nick.shields@otago.ac.nz (N.J.S.); 2 Department of Anatomy, Brain Health Research Centre and Brain Research New Zealand, University of Otago, P.O. Box 913, Dunedin 9054, New Zealand; 3 Faculty of Pharmacy, The University of Sydney, Sydney, NSW 2006, Australia; thomas.balle@sydney.edu.au (T.B.); mary.collins@sydney.edu.au (M.C.)

**Keywords:** stroke, inflammation, nicotinic, nicotinic acetylcholine receptor agonist, immune response, myeloid cells

## Abstract

Stroke is one of the leading causes of death and long-term disability, with limited treatment options available. Inflammation contributes to damage tissue in the central nervous system across a broad range of neuropathologies, including Alzheimer’s disease, pain, Schizophrenia, and stroke. While the immune system plays an important role in contributing to brain damage produced by ischemia, the damaged brain, in turn, can exert a powerful immune-suppressive effect that promotes infections and threatens the survival of stroke patients. Recently the cholinergic anti-inflammatory pathway, in particular its modulation using α7-nicotinic acetylcholine receptor (α7-nAChR) ligands, has shown potential as a strategy to dampen the inflammatory response and facilitate functional recovery in stroke patients. Here we discuss the current literature on stroke-induced inflammation and the effects of α7-nAChR modulators on innate immune cells.

## 1. Introduction

Stroke is one of the leading causes of death and morbidity in developed nations. Restricted blood supply following a stroke leads to a localised depletion in energy and oxygen, ultimately resulting in cell death within the affected area [1]. These dying cells secrete danger signals that stimulate an inflammatory response intended to support healing but which is, in most cases, excessive and exacerbates neural damage to impede recovery. This post-stroke inflammatory response is mediated by local innate immune cells in the brain (resident microglia) and by other immune cells that later enter the brain from the bloodstream. As a consequence of excess inflammation, recovery following a stroke can be severely hampered due to ineffective nerve cell repair.

The inflammatory process is stimulated by danger signals, which include fragments of cells undergoing cell death and factors secreted by damaged cells, such as adenosine triphosphate (ATP), uric acid, and reactive oxygen species (ROS) [2,3]. Even though danger signals are diverse in nature, they converge on similar pathways in local immune cells to stimulate the production and secretion of pro-inflammatory cytokines. These pro-inflammatory cytokines activate local microglial cells, induce the migration of blood-borne immune cells to the infarct area, and influence systemic immune responses. Paradoxically, the excessive inflammatory response in the brain induces a state of immune-suppression in the periphery, putting stroke patients at risk of fatal secondary infections [4]. The mechanistic relationship between the initial inflammatory response in the brain and the immune-suppression in the periphery observed in the weeks after stroke has long been unclear. Only recent evidence suggests that pro-inflammatory cytokines released in the brain stimulate the expansion of an immune-suppressive cell population that suppresses both innate and adaptive immune responses [5].

Inflammatory reactions are usually self-limiting and resolve, driven by the secretion of inhibitory molecules within the immune system. Interestingly, recent research has shown that the autonomic nervous system also controls inflammation via neural circuits that affect immune cells [6]. One of these brain-immune connections is the cholinergic anti-inflammatory pathway, which senses inflammation via peripheral nerves and suppresses the production of pro-inflammatory cytokines [7]. Remarkably, this interaction between the brain and the immune system relies on neurons sensing the presence of cytokines (immune-modulating agents). Upon sensing the presence of pro-inflammatory cytokines, such as interleukin (IL)-1β and tumour necrosis factor (TNF)-α, neurons release the neurotransmitter acetylcholine (ACh) which binds to nicotinic acetylcholine receptors (nAChR) on peripheral immune cells. Notably, activation of the α7-nAChR has recently been shown to improve functional recovery after stroke by limiting harmful post-stroke inflammation [8].

## 2. Overview of Nicotinic Acetylcholine Receptors (nAChRs) with a Focus on the α7-Subunit

Neuronal nAChRs are allosteric transmembrane proteins that are assembled from one or more α-subunits (α1–α10) either alone or together with one or more β-subunits (β1–β4) [9,10,11]. Each conformation of the nAChRs has a unique function, pharmacological profile, and expression pattern, which makes it possible for subtype-selective compounds to have distinct therapeutic applications with a restricted set of side effects [12,13]. nAChRs are widely distributed throughout the central nervous system, where they participate in a variety of physiological responses, like learning, memory, locomotion, and attention, among others. For the purpose of this review, we will focus on the α7-nAChR, which is one of the most abundantly-expressed and widely-distributed nAChRs throughout the brain [11,13,14]. In the brain, α7-nAChRs are expressed on neurons and non-neuronal cells, including astrocytes, microglia, oligodendrocytes, and endothelial cells [15,16,17]. Previous studies have highlighted that α7-nAChR modulators can minimise the extent of cell death [18,19] and enhance synaptic plasticity [20], making α7-nAChRs an ideal therapeutic target for several neurological diseases including depression, Parkinson’s disease, schizophrenia and Alzheimer’s disease [21]. Furthermore, positive α7-nAChR modulators are potent inhibitors of inflammation [22] and have been suggested as promising candidates for the treatment of inflammatory diseases, such as inflammatory bowel disease, arthritis, asthma, and obesity. α7-nAChR agonists have the advantage of selectively stimulating the α7-subunit, circumventing side effects caused through general activation of nicotinic receptors. This has led to the establishment of several clinical trials investigating the effectiveness of α7-nAChR agonists, predominantly for the treatment of cognitive disorders, such as dementia and schizophrenia [23,24]. Even though the side effect profile of selective α7-nAChRs is favourable in comparison to non-selective agonists, such as nicotine, it remains to be determined if the observed pharmacological effects are as potent. The mechanisms by which α7-nAChR agonists inhibit inflammation and promote an anti-inflammatory environment will be discussed in more detail herein.

## 3. An Inflammatory Reflex Shapes Immune Responses Post-Stroke via Activation of α7-nAChRs

The vagus nerve (cranial nerve X) is the longest of the cranial nerves and its main function is to regulate the parasympathetic nervous system. Previous studies have shown that stimulation of the vagus nerve can reduce inflammation, both in peripheral lymphoid organs as well as in the brain [6,25]. The complex interactions between the nervous system and the immune system that constitutes this “inflammatory reflex” are still not completely understood [26]. However, it is clear that the anti-inflammatory effects observed after vagal stimulation are mediated via the activation of α7-nAChRs found on innate immune cells [27]. α7-nAChRs can be found in tissues throughout the central nervous system (CNS) and are also abundantly expressed by immune cells [9]. Activation of α7-nAChRs on immune cells has been shown to minimise the production of pro-inflammatory cytokines [22], while, importantly, not abating the secretion of anti-inflammatory cytokines that promote the resolution of inflammation [28]. Innate immune cells are an attractive target for the treatment of stroke, as they are responsible for inducing sterile inflammation (inflammation not caused by microorganisms). This is mediated through the secretion of high amounts of pro-inflammatory cytokines, including IL-1β, IL-6, IL-8, IL-12 and TNF-α, by these cells. Elucidating the complex crosstalk that exists between immune responses and the nervous system may provide a broad new range of targets for novel therapies, aimed at reducing inflammation, and improving recovery from stroke.

In the following sections, we will discuss the role of innate immune cells in the development of sterile inflammation after stroke, focusing on how this impairs recovery and shapes the generation of an immune-suppressive cell population that further compromises the health of stroke patients. We will discuss the involvement of these recently-discovered immature myeloid cells in suppressing immune responses during the sub-acute phase (days) after stroke and how this cell population could potentially be targeted therapeutically. In addition, we will discuss the potential of α7-nAChR agonists in affording neuroprotection, resolving inflammation and aiding in functional recovery.

## 4. Inflammatory Response of the Immune System Post-Stroke

Innate immune cells are considered the body’s first line of defence, providing protection from potentially harmful pathogens. They also have a crucial role in both initiating and advancing repair processes through the induction of inflammation. Whether this role is associated with improved recovery or further tissue damage in stroke patients depends partly on the size of the initial infarct and the ensuing inflammatory response. Post-ischemic inflammatory cascades are initially prompted by a lack of oxygen, with the resulting metabolic failure leading to uncontrolled cell death [29]. The membrane of cells undergoing uncontrolled cell death typically disintegrates and releases cellular content and organelles into the surrounding tissue [30]. The released cellular contents are then recognised as danger signals by the innate immune system, namely specialised phagocytic cells, and are crucial for initiating inflammation [31].

The CNS has its own phagocytic innate immune cells, called microglia, that share similarities to peripheral macrophages. These long-lived microglial cells, which develop from haematopoietic progenitors that take up residency in the CNS during early embryogenesis, can self-renew, and proliferate in response to stimuli [32]. Microglia are the first immune cells to detect and immediately respond to cellular damage caused by stroke, supporting the clean-up of the infarct area [33]. This is possible due to a wide variety of pattern recognition receptors (PRRs), which are expressed ubiquitously on the surface and in intracellular compartments of innate immune cells [34]. PRRs bind pathogens and danger signals, also known as damage-associated molecular patterns (DAMPs), with high affinity and allow for the rapid detection of changes in the tissue environment [35]. The binding of DAMPs to PRRs and the subsequent activation of intracellular signalling pathways within microglia increase the expression of co-stimulatory cell surface molecules and, finally, the production of pro-inflammatory cytokines by these cells [36]. Both of these processes are necessary to attract and stimulate adaptive immune cells, called lymphocytes, which are capable of recognising antigens and forming memory cell populations. Long-lived memory B- and T-lymphocytes provide long-lasting immune protection and respond rapidly upon secondary encounter with their cognate antigen [37,38]. However, in the case of brain injury where adaptive immunity is not required, the secretion of pro-inflammatory cytokines may tip the balance of the immune response from tissue healing and resolution to destruction. In contrast to innate immune cells in the periphery, microglia remain chronically activated even in the absence of initial DAMPs [39,40]. This is associated with the continued secretion of pro-inflammatory factors that drive chronic neuroinflammation and, thus, contributes to delayed recovery in stroke patients [41]. Thus, microglia are damaging in the sub-acute and recovery phase, whereas the initial inflammatory response mediated by these cells is vital in the acute phase following stroke. This illustrates the biphasic dynamics of inflammatory signalling post-stroke, which can be protective or deleterious depending on the context. For example, the secretion of growth factors and matrix metalloproteinases (MMPs) are vital for tissue re-construction and vascularisation processes in the sub-acute and recovery phase of stroke, while being detrimental in the acute phase [42]. In addition to these observations, many other studies [29] emphasise the biphasic role of inflammation in stroke recovery, highlighting the need to carefully tailor anti-inflammatory strategies in order to maximise stroke recovery.

In addition to the local response, pro-inflammatory cytokines secreted by microglia reach the blood stream and attract innate immune cells from within the circulatory system to infiltrate the damaged tissue, which is facilitated by the breakdown of the blood-brain barrier [43]. Whilst blood-derived innate immune cells can help with the clearance of damaged tissue in the area of infarction, they can also exacerbate tissue damage by producing more pro-inflammatory cytokines and ROS [36].

Consistent with this, recent findings have shown that inflammation impairs sensory learning and cortical plasticity after stroke [44]. In addition, the depletion of T-cells, especially of CD4^+^ T-cells, results in increased neurogenesis and accelerates functional recovery [45]. As both synaptic plasticity and neurogenesis are crucial for recovery after stroke [46,47], specific targeting of these processes may yield effective therapies. Notably, positive α7-nAChR modulators appear to be ideal candidates as they can both enhance synaptic plasticity [20] and neurogenesis [48]. However, further studies are required to validate α7-nAChR modulators as an effective treatment option for stroke before they can be tested in a clinical setting.

### 4.1. Early Innate Immune Responses Post-Stroke

The activation of microglia through DAMPs and the release of pro-inflammatory cytokines leads to the expansion of the local microglial cell population and attraction of blood-borne innate immune cells to the site of injury [49]. The infiltrating cells are generally thought to be macrophages of myeloid origin based on their high expression of CD45 (lymphocyte common antigen) and CD11b (cluster of differentiation molecule 11b), whereas microglia exhibit a low expression of CD45 and can be distinguished from macrophages on this basis [50]. Furthermore, in parasymbiosis experiments where two mice are surgically joined in order to share blood, researchers have been able to distinguish infiltrating blood-derived immune cells from local microglial populations [33,51]. These experiments demonstrated that circulating cells are able to enter the CNS post-stroke and that resident microglia are the main cell population that expands in response to injury [51]. Further studies have elucidated the importance of resident microglia in the immediate response following stroke, but less is known regarding the role of infiltrating myeloid cells in this context [50,52].

#### 4.1.1. Myeloid Cells

Myeloid cells (monocytes, macrophages, dendritic cells, granulocytes, and erythrocytes) and lymphoid cells (T- and B-cells, natural killer cells) arise from multipotent haematopoietic stem cells in the bone marrow. The differentiation of these cells into specialised subsets depends on the environmental stimuli they are exposed to (e.g., growth factors and cytokines) and/or their lineage origin [53,54]. Myeloid cells are vital for the response to and clearing of infections, wound healing, and tissue homeostasis. They respond rapidly to tissue damage and can attract adaptive immune cells, such as T- and B-cells, to the site of injury. In damaged brain tissue following stroke, phagocytic myeloid cells play a key part in tissue repair and re-organisation, facilitating the clearance of necrotic cells and debris. However, despite their crucial roles in the healing process, myeloid cells can also amplify the inflammatory response and contribute to secondary tissue damage [55].

Profiling the temporal dynamics of infiltrating immune cells has revealed a massive influx of phagocytic cells, such as neutrophils, monocytes, dendritic cells (DCs, CD11c^+^ CD11b^+^) and macrophages (CD11c^−^ CD11b^+^), into the CNS around day three, post-stroke [43]. Depending on environmental stimuli, such as cytokines and inflammatory factors, monocytes circulating in the blood can migrate to injured tissues and give rise to tissue-resident macrophages and DCs. As different populations of monocytic cells share similarities in the expression of cell surface markers, the identification of specific monocytic cell populations has been challenging [56]. For example, the commonly-investigated cell surface markers CD11c and CD11b are interchangeably expressed by macrophages and DCs, as well as immature progenitor cells that egress from the bone marrow after trauma, injury, or infection [57].

Understanding what factors activate myeloid cells and disposes their function from pro-repair to pro-inflammatory and, hence, is detrimental, requires further investigation. The field of flow cytometry has advanced rapidly over the last decade and offers tremendous power for the identification of cell populations using a variety of molecular markers. The analysis of several molecular markers at once enables greater understanding of the phenotypic and functional characteristics of specific cell populations, providing the ability to better define their role in stroke pathology. With an improved understanding of the changes in cell populations, it may be possible to target myeloid cells using specific modulators to improve functional recovery in patients.

#### 4.1.2. Immature Myeloid Cells

The role of immature myeloid progenitor cells in stroke has only just started to be investigated and remains somewhat poorly understood. These cells originate from the bone marrow, where their development is inhibited in response to an inflammatory stimulus, such as those occurring in stroke, cancer, trauma, sepsis, and autoimmune diseases. In these circumstances, pro-inflammatory cytokine release arrests myeloid stem cell differentiation, leading to the expansion of immature myeloid cells which then migrate into the circulation [57]. While mature macrophages and DCs initiate and promote innate and adaptive immune responses, these immature counterparts have been found to have opposing roles and are immune-suppressive [57]. As a result, they have been termed “myeloid-derived suppressor cells” (MDSCs) [58]. Liesz and colleagues demonstrated that immature myeloid progenitor cells egress from the bone marrow in response to DAMPs, such as high-mobility group box 1 (HMGB1), secreted as a consequence of stroke [5]. This release of MDSCs from the bone marrow is beneficial following acute trauma, as they restrain excessive and potentially harmful immune responses in healthy individuals [59] and promote repair mechanisms, such as angiogenesis [60]. In other diseases, such as cancer where a potent immune response is beneficial, MDSCs can be detrimental and facilitate disease progression by suppressing anti-cancer immune responses [61].

Recently, two major subsets of MDSCs have been identified; granulocytic and monocytic MDSCs, which differentiate from a granulocytic and a monocytic precursors, respectively, in bone marrow [62,63]. The granulocytic subset shows phenotypic and morphological similarities to neutrophils and can be identified by high expression of CD11b and lymphocyte antigen Ly6G, whereas monocytic MDSCs are commonly characterised by high CD11b and Ly6C expression [63,64]. Both MDSC subsets are able to suppress T-cell responses, impair lymphocyte trafficking, induce regulatory T-cells, and skew innate immune cells towards an anti-inflammatory phenotype [61].

Of significance for stroke recovery, MDSCs promote vascularisation by secreting MMP-9 and 13 [60,65]. The biology and function of MDSCs is too broad and complex for the scope of this review, however, we refer the reader to these reviews for further details [57,61,65]. It should be noted that cell surface markers commonly used to study the phenotype of myeloid cells infiltrating the CNS post-stroke, such as macrophages, neutrophils, DCs and monocytes, are also expressed by MDSCs. Consequently, there has most likely been incorrect identification of infiltrating myeloid cells in the past. Therefore, detailed analysis and identification of cell populations using extensive flow cytometric markers should be employed to further elucidate the true phenotypic characteristics of infiltrating myeloid cells.

### 4.2. Infection Post-Stroke

Ischemic stroke is confounded by conditions such as atherosclerosis, diabetes, and infection, all of which alter peripheral inflammatory processes with a concomitant impact on stroke outcome. Furthermore, stroke commonly results in peripheral infections that can impede recovery. It has been reported that 23%–65% of patients who have a stroke will subsequently suffer from an infection, which is also associated with increased mortality and poor patient outcome [66]. Bacteria that cause these infections further stimulate the immune system to produce pro-inflammatory cytokines, which in turn contribute to secondary brain damage [29]. The rapid development of inflammation is one of the key elements of stroke pathophysiology and recent studies have demonstrated that modulation of early inflammatory responses improves recovery and outcomes after stroke [67,68]. Furthermore, the suppression of inflammation post-stroke as a therapeutic approach offers the unique advantage of prolonging the therapeutic window and enables the treatment of patients who do not qualify for thrombolysis. However, the pharmacological manipulation of inflammatory responses has to be carefully balanced as it may compromise tissue repair mechanisms. One novel and selective mechanism for dampening the pro-inflammatory cytokine response during early post-ischemic stroke is the activation of the α7-nAChR [68].

**Figure 1:**
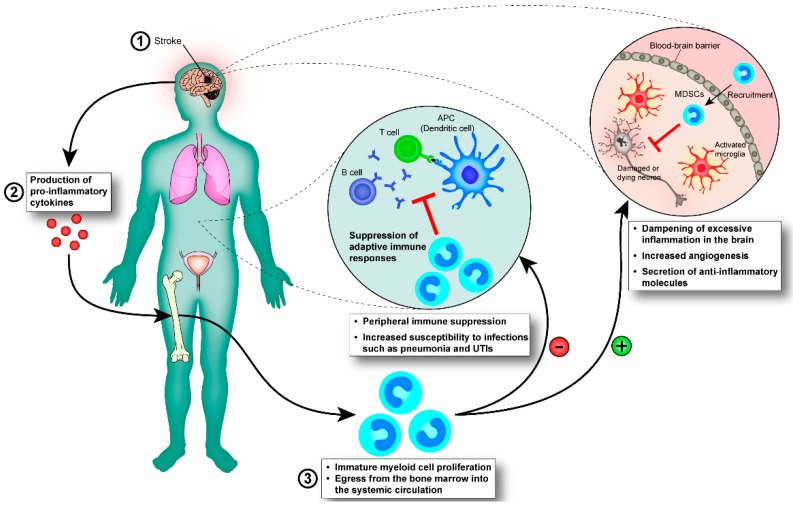
Immature myeloid cells mediate immune-suppression post-stroke. (**1**) Stroke is followed by (**2**) the production of pro-inflammatory cytokines, which reach the bone marrow via the blood-stream (**3**) where they stimulate the expansion of immature myeloid cells and partially block their maturation into granulocytes, macrophages, and DCs. The immature myeloid cells (also known as MDSCs) migrate along a chemokine/cytokine gradient to secondary lymphoid organs and to the infarction area where they exert anti-inflammatory effects on other cell populations. (−) Immature myeloid cells are able to suppress innate and adaptive immune responses in the periphery, exposing stroke patients to an increased risk of acquiring infections, such as pneumonia and urinary tract infections (UTIs), which impede recovery and can potentially be life-threatening. (+) We hypothesise that immature myeloid cells travelling from the bone marrow to the brain exert protective effects there. Immature myeloid cells are highly phagocytic and would help clearing the infarct area of dead cells, and they are known to dampen inflammation through the secretion of cytokines that skew innate immune cells towards an anti-inflammatory phenotype. Furthermore, they are able to induce angiogenesis through the secretion of MMPs, which facilitates re-vascularisation. All of these effects would be beneficial, acting to limit excessive inflammation and promote healing. MMPs, matrix metalloproteinases.

Taking into account the aforementioned features of MDSCs, it is possible that they represent a physiological response that functions to control and dampen inflammation post-stroke (Figure 1). MDSCs are highly phagocytic [69], they prevent adaptive immune responses [61], support tissue re-organisation via MMP-mediated angiogenesis [70] and, in general, secrete anti-inflammatory factors that break the destructive cycle of chronic inflammation. Unfortunately, the actions of MDSCs are not limited to the CNS where they are desirable, and as a consequence, they can also affect immune responses occurring in the periphery.

Infections acquired in the days to weeks following a stroke are the major cause for mortality in stroke patients [71], as they are unable to resolve usually non-life-threatening infections, such as respiratory and urinary tract infections. Liesz and colleagues have provided what appears to be the first evidence that MDSCs might be responsible for inducing immune-suppression post-stroke [5]. Further, evidence to support the theory that MDSCs are responsible for the observed immune-suppression after stroke comes from the analysis of gene expression in circulating neutrophils and monocytes in humans [72]. The expression of arginase 1 (expressed by monocytic MDSCs), S100A9 (induces generation and expansion of MDSCs), and MMP-9 (secreted by MDSCs), were found to be amongst the genes that showed the highest increase in expression post-stroke [72]. These findings suggest that MDSCs may circulate in the bloodstream after stroke and shape systemic inflammatory responses.

### 4.3. Pattern Recognition Receptors in Stroke

Even though innate immune cells in the brain and periphery have different phenotypes, they all sense DAMPs through PRRs. In the context of stroke, the toll-like receptors (TLRs) have been the most extensively studied PRRs due to their ability to activate microglia and peripheral innate immune cells. TLRs are a family of transmembrane proteins that are located primarily on the cell surface (TLR 1, 2, 4, 5, 6 and 11) or in intracellular compartments (TLR 3, 7, 8 and 9) [34]. The downstream effects of TLR-signalling include activation of the adapter molecules myeloid-differentiation factor 88 (MyD88) or TIR-domain-containing adapter-inducing interferon-β (TRIF) [73]. Stimulation of these pathways results in the activation of pro-inflammatory signalling cascades that mainly involve the transcription factor nuclear factor (NF)-κB. Upon stimulation of PRRs and downstream adaptor proteins, NF-κB translocates to the nucleus and induces the transcription of genes that code for pro-inflammatory cytokines. Even though stimulation of some PRRs (such as the TLR4), and the subsequent production of pro-inflammatory cytokines has been linked to increased infarct size and worse clinical outcome in stroke patients, the role of NF-κB activation as a consequence of stroke remains controversial [74,75]. Unexpectedly, recent studies suggest that activation of TLR4 is an important aspect of microglial phagocytosis and correlates with increased neurogenesis after stroke [76,77].

TLRs are activated by a multitude of DAMPs, such as heat shock proteins, nucleic acids [78], purine metabolites (e.g., ATP [2], uric acid [3]), and HMGB1 [79]. In addition, activated microglia are able to further propagate inflammatory responses by secreting ligands for the TLR4. One of these recently identified endogenous ligands, carbohydrate-binding lectin galectin-3, activates neighbouring immune cells in a paracrine manner [80]. Depending on their nature, DAMPs bind to specific TLRs, which in turn, influences the type of cytokines secreted. For example, signalling involving the adapter molecule TRIF, through the binding of double-stranded RNA to TLR3, stimulates the secretion of type I interferons [81]. In stroke, administration of an interferon γ (IFN-γ) neutralising antibody has been shown to decrease infarct size, as well as the number of CD3^+^ cells that infiltrate the infarct [82]. The ambivalence of TLR signalling in stroke needs further elucidation to clearly identify molecular targets that convey neuroprotection and/or enhance recovery when modulated at a delay post-stroke.

**Figure 2:**
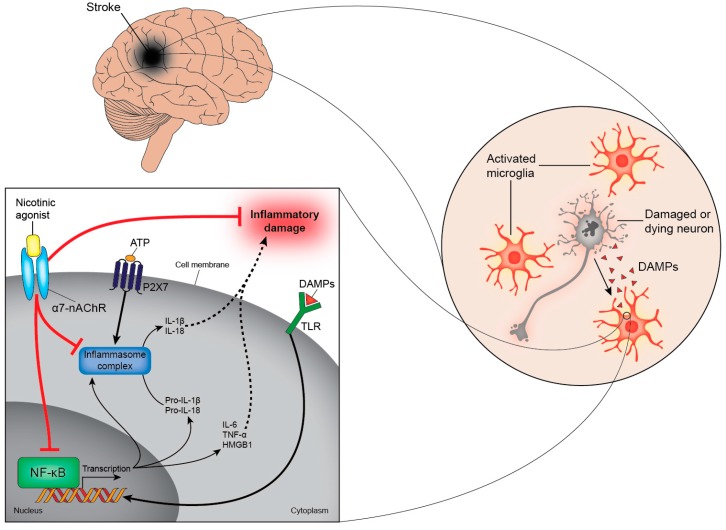
Activation of α7-nAChRs reduces inflammation. Damaged and dying cells in the brain release DAMPs after a stroke. DAMPs can bind to several receptors, including TLRs, cytokine receptors, and the receptor for advanced glycation end products (RAGE). Ligation of these receptors activates NF-κB to translocate to the nucleus (bold arrow) where it induces the transcription of genes that code for pro-inflammatory cytokines, such as IL-6, IL-8, TNF-α and HMGB1. In addition, activation of NF-κB stimulates the production of the immature forms of IL-1β and IL-18 (pro-IL-1β and pro-IL-18, respectively). These biologically-inactive cytokines are cleaved to their active form by inflammasomes and are subsequently secreted from the cell (dashed arrows). These intracellular multi-protein complexes assemble in response to NF-κB stimulation and require a second stimulus (such as ATP) to become activated. α7-nAChR agonists interfere with these pathways and prevent the secretion of pro-inflammatory cytokines by inhibiting NF-κB and inflammasome activation (bar-headed red lines). α7-nAChRs, α7-nicotinic acetylcholine receptor; DAMPs, damage-associated molecular patterns; TLRs, toll-like receptors; NF-κB, nuclear factor kappa B; IL, interleukin; TNF-α, tumour necrosis factor-α; HMGB1, high-mobility group box 1; ATP, adenosine triphosphate.

Instead of targeting individual TLRs to prevent the synthesis of pro-inflammatory cytokines, a more elegant solution might be to inhibit the common endpoint for all TLRs; the activation of NF-kB. Inhibiting NF-kB is one of the mechanisms by which α7-nAChR agonists reduce inflammation [83]. It has been demonstrated that activation of α7-nAChRs on neural microglia, peripheral macrophages, and monocytes lowers the secretion of pro-inflammatory cytokines, resulting in reduced neuroinflammation (Figure 2) and improved outcomes in experimental models of stroke [84,85,86]. Additionally, α7-nAChR agonists stimulate the Janus kinase 2-Signal transducer and activator of transcription 3 (JAK2-STAT3) pathway and, in turn, the secretion of anti-inflammatory cytokines, such as IL-10 [87,88].

The release of the cytokine-like protein, HMGB1, from necrotic cells in the infarct area activates microglia to a pro-inflammatory state, inducing the synthesis of cytokines and other inflammatory molecules that contribute to further tissue damage [79,89]. Although a reduction of HMGB1 production in the acute phase improves stroke recovery [90], it is vital for the induction of repair processes in the sub-acute phase. This involves the promotion of angiogenesis through a VEGF-dependent mechanism [91], the recruitment of stem cells [92] and the support of neurovascular remodelling processes mediated by activated astrocytes [93]. Studies have also reported that HMGB1 is inhibited in the presence of α7-nAChR agonists [83].

Interestingly, recent evidence indicates that inflammation can down-regulate the expression of α7-nAChRs in the brain, which is associated with the accumulation of β-amyloid and the development of Alzheimer’s disease [94]. This evidence could help explain the increased prevalence of stroke patients developing dementia and also suggests that inflammation is one of the primary causes for dementia [94,95]. Nicotine, through its binding to α7-nAChRs, has been shown to prevent and reverse the accumulation of β-amyloid in the brain, highlighting a potential new therapeutic target for preventing the development of post-stroke dementia [96].

These findings highlight the fact that mediators of inflammation may have biphasic roles in stroke recovery and even though counteracting inflammatory processes in the acute phase is beneficial, it may be detrimental later on. Therapeutic approaches using α7-nAChR agonists, for example, should be carefully tailored to support not only the dampening of inflammatory cascades but to also allow the enhancement of tissue repair processes.

This seems a particularly promising approach as nAChR agonists up-regulate the cell surface expression of the receptors, whereas most drug therapies either cause the receptors to become down-regulated or internalised following sustained exposure to their ligands [97]. Therefore, the activation of α7-nAChRs may present a unique system to decrease inflammation in the acute phase after stroke and to also prevent secondary damage.

### 4.4. Purinergic Signalling in Stroke

**Figure 3:**
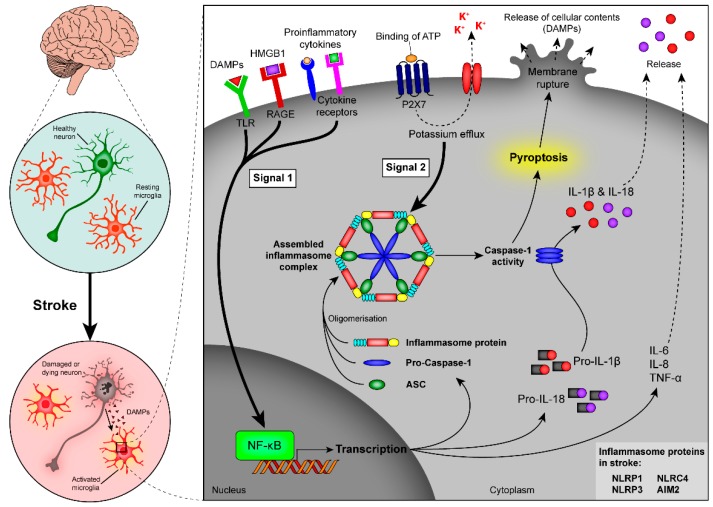
Induction of inflammasome activity and pyroptosis after stroke. Signalling through PRRs results in NF-κB activation, which is associated with up-regulated transcription of inflammasome components (inflammasome proteins, pro-caspase-1, and ASC) and the immature cytokines pro-IL-1β and pro-IL-18, as well as an increased production of pro-inflammatory cytokines, such as IL-6, IL-8, and TNF-α (**signal 1, bold arrow**). A second signal (**signal 2, bold arrow**) leads to oligomerisation of the inflammasome components and activates the inflammasome complex. Signal 2 can, for example, occur through binding of ATP to its P2X7 receptor. As a result of inflammasome assembly and activation, caspase-1 gets activated and cleaves the inactive cytokines pro-IL-1β and pro-IL-18 to their active form, which are released from the cell (dashed arrows). Caspase-1 is also an important mediator of pyroptosis, a highly-inflammatory form of cell death, which results in the swelling and bursting of cells with the subsequent release of cell content into the extracellular space. PRRs, pattern recognition receptors; NF-κB, nuclear factor kappa B; ASC, apoptosis-associated speck-like protein containing a CARD; IL, interleukin; tumour necrosis factor-α; ATP, adenosine triphosphate.

nAChR agonists can modulate the signalling of other important endogenous DAMPs, such as ATP. ATP acts as a universal stress signal, detected by purinergic receptors on a multitude of immune and non-immune cells [98]. ATP is recognised by G-protein coupled P2Y and P2X receptors, which are ligand-gated ion channels that regulate the influx and efflux of cations [99]. Interestingly, ATP not only acts as a DAMP but, when secreted at low concentrations, can induce the controlled digestion of dying cells, which is important to promote the clearance of injured tissue without the induction of chronic inflammation [100]. In contrast, the sudden secretion of high concentrations of ATP by dying cells after a stroke triggers a pro-inflammatory response [101,102]. High ATP concentrations activate intracellular PRRs, which are part of multi-protein complexes termed “inflammasomes”. Inflammasomes contain an intracellular PRR, such as the NOD-like receptor (NLRs), which recognise DAMPs [103]. DAMPs are taken up by phagocytosis or enter the cytosol via channels in the cell membrane initiated by pore-forming molecules [104]. ATP activates the inflammasome via binding to P2X receptors, which results in the efflux of potassium ions [105]. Independent of the type of DAMP, an initial priming step is necessary to induce the transcription of pro-IL-1β and the assembly of the inflammasome complex (Figure 3). Molecules that provide the first signal include ligands for TLRs, NLRs, RIG-1-like receptors (RLRs), and certain cytokine receptors [105].

So far, several nAChR agonists have been identified that inhibit the ATP-dependent activation of inflammasomes. These molecules are mainly endogenous ligands, such as acetylcholine, choline, and modifications thereof [106,107]. It has been proposed that binding of the endogenous ligand acetylcholine modulates ATP-induced changes resulting in inhibition of inflammasome activation. Lu and colleagues demonstrated that acetylcholine could prevent the ATP-induced mitochondrial perturbations and thus inflammasome activation by binding to the α7-nAChR in mitochondria [107]. Furthermore, Hecker and colleagues demonstrated that acetylcholine interrupted ATP-induced ion currents and thereby prevented inflammasome activation [106]. Both of these results indicate that nAChR ligands interfere with ATP signalling. It remains to be determined if inflammasome activation can also be blocked if induced by different DAMPs.

The assembly of inflammasomes is required for the processing and secretion of pro-inflammatory cytokines, such as IL-1β, which has been shown to contribute to pathological diseases such as cancer, diabetes, dementia, and stroke [108,109]. IL-1β has pleiotropic functions in the inflammatory response, that include the stimulation of non-haematopoietic cells to secrete chemokines that promote the infiltration of neutrophils, increasing the expression of adhesion molecules, and induction of cyclooxygenase 2 (COX-2) [110] and inducible nitric oxide synthase (iNOS) gene expression [111]. Together, these mechanisms trigger an influx of inflammatory cells from the circulation into regions of tissue damage where they impair the processes associated with tissue remodelling [112].

### 4.5. Role of Inflammasomes in Stroke

It has been known for some time that inhibition of IL-1β signalling, downstream of inflammasome activation, is an effective means to decrease the size of infarction and improve function after stroke in experimental animal models [109,113,114]. Results from randomised human clinical trials look promising and indicate that administration of IL-1β antagonists in the first 72 h post-stroke dampens circulating pro-inflammatory cytokine levels in both blood and cerebrospinal fluid—whether this will translate into beneficial therapeutic outcomes has yet to be confirmed [115,116].

The type of inflammasomes involved in the inflammatory response and, thus, in the production of IL-1β and IL-18 after stroke is slowly being unravelled. The NLRP1 inflammasome was the first inflammasome identified to form and be activated post-stroke [117]. The NLRP1 complex consists of the cytoplasmic NACHT leucine-rich repeat protein 1 (NLRP1), the adaptor molecule apoptosis-associated speck-like protein containing a CARD (ASC), and the pro-inflammatory caspases-1 and 5 [118]. After injury to the brain, the NLRP1 inflammasome mainly assembles in neurons, astrocytes, and microglia [117,119]. On the other hand, the NLRP3 inflammasome, which is commonly associated with the sensing of necrotic cell death, is predominantly expressed in microglia and endothelial cells [120]. Pharmacological targeting of either the NLRP1 or NLRP3 inflammasomes has been shown to reduce innate immune responses and reduce infarct size post-stroke [114,117,119,120]. However, a recent study implied several other inflammasomes might also play a role in the pathology of stroke [121]. In addition, Denes and colleagues highlighted the adaptor molecule, ASC, as being a crucial regulator in the inflammatory response after stroke, whereas inhibition of the other components that form the NLRP3 inflammasome complex did not improve stroke outcome [121]. In particular, it was shown that deficiency in the NLRC4 (CARD domain containing 4) or AIM2 inflammasomes (absent in melanoma 2) was beneficial in reducing inflammation-related tissue damages; both of these inflammasomes require ASC to recruit and activate caspase-1, which cleaves the immature cytokines into their biologically active form [121,122]. These recent reports demonstrate the need to clearly identify if all inflammasomes containing an ASC contribute to tissue damage post-stroke, as this could become a likely candidate for targeting future drug treatments.

The recent findings that several inflammasomes are involved in the inflammatory response post-stroke are particularly interesting in the context of cell death (Figure 3). A recently identified caspase-1-dependent form of cell death, also known as pyroptosis, is characterised by rapid cell membrane rupture due to swelling, followed by the release of pro-inflammatory content into the extracellular space [123]. Cell death due to pyroptosis is highly inflammatory and is associated with an increase in cell size and the cleavage of genomic DNA [124]. Double-stranded host DNA segments act as DAMPs and can directly bind to TLR9 and/or the AIM2 inflammasome, which leads to further activation of caspase-1 and subsequent release of IL-1β and IL-18 [122,125].

The activation of inflammasomes through the sensing of DAMPs in microglia, endothelial cells, astrocytes, and neurons, is responsible for this detrimental inflammatory cycle. The assembly of inflammasomes leads to the activation of caspase-1 which, in turn, instigates pyroptotic cell death. It seems inevitable that the release of pro-inflammatory content into the extracellular space due to pyroptotic cell death triggers the activation of other inflammasomes, such as the AIM2 inflammasome, that have been implicated in the inflammatory response post-stroke [121], fuelling an excessive inflammatory response. As such a process propagates a cycle of cell death, compounds that can halt this process would likely result in minimised cell death and smaller infarct size. In accordance with this concept of caspase-1-dependent cell death is the finding that caspase-1 deficient mice have been found to have smaller ischemic lesions [126]. Recent evidence also suggests that inflammasome and caspase-1 activation in endothelial cells reduces angiogenesis after stroke [127]. Angiogenesis is an important repair mechanism after stroke—it not only stimulates re-vascularisation of the damaged tissue but also attracts newly born neurons to migrate to the site of injury where they co-localise with newly formed blood vessels [128].

## 5. Conclusions

Inflammatory responses in the acute phase after stroke impair functional recovery but contribute to tissue repair and re-modelling processes in the sub-acute phase. The biphasic nature of inflammation has become evident in recent years and this will help to optimise anti-inflammatory stroke treatments. A variety of molecules have been shown to effectively inhibit inflammatory signalling cascades at different levels, which may translate into a reduction of immune-suppression observed in the weeks after stroke. Particularly interesting are the versatile effects of α7-nAChR agonists on innate immune cells. The observation that they suppress inflammation and potentially minimise the risk of stroke-associated morbidities, through the up-regulation of α7-nAChRs, make them promising candidates for further study. In addition, the more thorough characterisation of infiltrating immune cells after stroke will provide further information on how this can be achieved.

The discovery that MDSCs are the cells that convey immune suppression after stroke will greatly advance the field of stroke immunology. The phenotype, the mechanisms of action, and the role of MDSCs in disease progression have been systematically studied in the context of many other diseases, such as cancer and sepsis, which will aid our understanding of their role in stroke. Similar to the inflammatory process itself, MDSCs may be shown to play a biphasic role in stroke recovery. On the one hand, they limit inflammation and initiate vital tissue re-modelling processes but they also mediate an immune-suppressive state in the periphery responsible for stroke fatalities. In conclusion, it appears crucial to finely balance the inflammatory response to sufficiently engage the immune system in tissue repair processes but to also prevent detrimental effects of excessive inflammation after stroke.
